# Prediction of postpartum prediabetes by machine learning methods in women with gestational diabetes mellitus

**DOI:** 10.1016/j.isci.2023.107846

**Published:** 2023-09-09

**Authors:** Durga Parkhi, Nishanthi Periyathambi, Yonas Ghebremichael-Weldeselassie, Vinod Patel, Nithya Sukumar, Rahul Siddharthan, Leelavati Narlikar, Ponnusamy Saravanan

**Affiliations:** 1 Populations, Evidence, and Technologies, Division of Health Sciences, University of Warwick, Coventry, UK; 2 Department of Diabetes, Endocrinology, and Metabolism, George Eliot Hospital, Nuneaton, UK; 3 School of Mathematics and Statistics, The Open University, Milton Keynes, UK; 4 Department of Computational Biology, The Institute of Mathematical Sciences, Chennai, India; 5 Department of Data Science, Indian Institute of Science Education and Research, Pune, India

**Keywords:** Endocrinology, Reproductive medicine, Female reproductive endocrinology, Computational bioinformatics

## Abstract

Early onset of type 2 diabetes and cardiovascular disease are common complications for women diagnosed with gestational diabetes. Prediabetes refers to a condition in which blood glucose levels are higher than normal, but not yet high enough to be diagnosed as type 2 diabetes. Currently, there is no accurate way of knowing which women with gestational diabetes are likely to develop postpartum prediabetes. This study aims to predict the risk of postpartum prediabetes in women diagnosed with gestational diabetes. Our sparse logistic regression approach selects only two variables – antenatal fasting glucose at OGTT and HbA1c soon after the diagnosis of GDM – as relevant, but gives an area under the receiver operating characteristic curve of 0.72, outperforming all other methods. We envision this to be a practical solution, which coupled with a targeted follow-up of high-risk women, could yield better cardiometabolic outcomes in women with a history of GDM.

## Highlights

• Personalized prediction model for accurate prediction of ppGI after GDM is feasible
• LR gave AUC of 0.72 compared to HGBC with balanced bagging which gave AUC of 0.7
• Proposed optimal cut-offs for LMICs for early risk stratification of women with ppGI

## Graphical abstract

**Figure:**
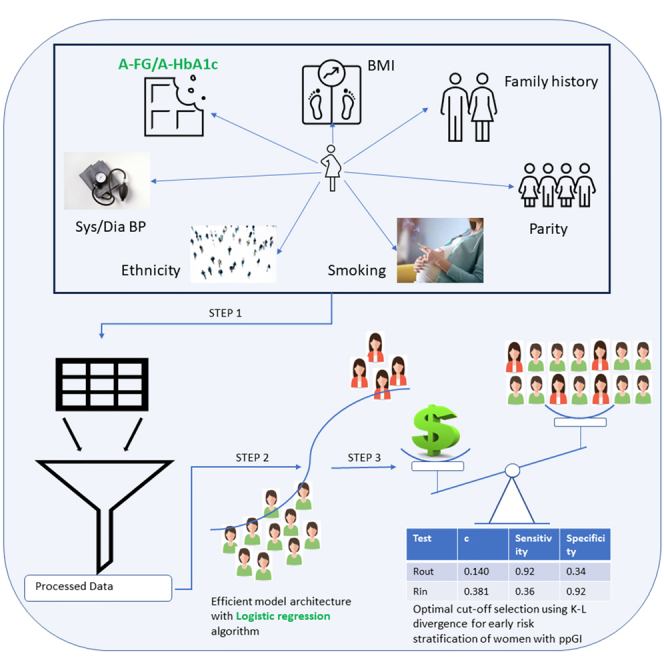

## Introduction

Gestational diabetes mellitus (GDM) is defined as any degree of prediabetes with onset or first recognition during pregnancy. Women diagnosed with GDM have up to 10-fold higher risk of Type 2 diabetes mellitus (T2DM) compared to those without GDM^1^ and their lifetime risk is around 60% for developing T2DM.^2^ In addition to T2DM, GDM women have a 2-fold higher risk of cardiovascular disease (CVD), at a younger age, and independent of intercurrent T2DM.^3^^,^^4^^,^^5^^,^^6^ GDM is associated with an increased risk of cardiovascular dysfunction, including rise in cardiovascular risk factors like blood pressure, and adverse changes in cholesterol and triglycerides.^7^ However, this risk is not the same for all women diagnosed with GDM.

There is some evidence that glucose levels during pregnancy are predictive of prediabetes.^8^^,^^9^ Retnakaran et al.^10^ have shown that the risk of dysglycamia at 12 weeks postpartum increases across the groups from normal glucose challenge test (GCT) and Normal Glucose Tolerance (NGT), to abnormal GCT and NGT, to gestational impaired glucose tolerance (GIGT), to GDM. This has been supported by other studies.^11^^,^^12^ Higher fasting glucose shows a high tendency of conversion to T2DM in the postpartum period^7^^,^^13^ and antenatal fasting glucose > 5.7 mmol/L is considered to be an important antenatal variable for the prediction of postpartum abnormal glucose metabolism.^14^

Along with glucose values in pregnancy, many studies have proposed the significance of gestational age at the time of diagnosis of GDM in predicting postpartum prediabetes.^15^^,^^16^ Specifically, women diagnosed at 24 weeks of gestation or earlier are at higher risk of having postpartum prediabetes.^17^ Similarly, the requirement of insulin therapy during pregnancy, ethnicity, gravidity, BMI, weight at the time of delivery, and neonatal weight are other factors that have been shown to be associated with the risk of prediabetes.^18^ While there is ample evidence of multiple factors being associated with T2DM onset in GDM-diagnosed women in general, there is no personalized risk score that can predict whether a specific GDM-diagnosed woman is likely to develop prediabetes or T2DM. Indeed, identifying women who are especially at high risk can help in implementing targeted, personalized interventions to delay and prevent the onset of T2DM and its future complications.

Artificial intelligence has begun to play a dominant role in healthcare, facilitating optimal decision-making as well as personalized treatment. Although Kumar et al.^19^ and Muche et al.^20^ have shown evidence of using machine learning for predicting progression of GDM to postpartum Type 2 diabetes, its use in the development of predictive models for T2DM onset is still in its nascent stages. Accurate prediabetes risk stratification at or before delivery for GDM women could assist policymakers and clinicians in specifically targeting those at the highest risk, especially in resource-constrained settings.

Postpartum screening is poor in many parts of the world as women have many competing interests on their time during this period.^21^^,^^22^ We and others have shown that women who miss postpartum screening had higher cardiometabolic risk factors.^23^^,^^24^ While dedicated healthcare administrators can improve the screening, this is still suboptimal. Therefore, a strategy that is personalized by identifying who is at risk of developing postpartum prediabetes/diabetes could help healthcare professionals for targeted education on the importance of screening for prediabetes/diabetes following a GDM pregnancy.

The primary aim of this paper is to investigate the predictive ability of the antenatal variables and derive a model for personalized prediction of prediabetes. We explored the use of logistic regression (LR) and tree-based machine learning algorithms for developing the prognostic model. We report our findings on a multi-ethnic retrospective cohort in the UK.

## Methods

### Data acquisition

A retrospective audit of electronic database records of postpartum screening at 6 to 13 weeks of women diagnosed with GDM, from January 2016 to December 2019, was conducted at an NHS trust hospital in the UK. GDM was diagnosed using NICE 2015 criteria.^25^ Complete data are available for 607 women for the following variables: age, height, weight, BMI, systolic and diastolic BP at booking, ethnicity, gravida, parity, smoking status, married status, employment status, gestational age at delivery, mode of delivery, birth weight, breastfeeding status, and biochemical variables such as antenatal fasting glucose (A-FG), antenatal postprandial glucose (A-PG), antenatal HbA1c (A-HbA1c), postpartum fasting glucose (P-FG), postpartum postprandial glucose (P-PG), and postpartum HbA1c (P-HbA1c). Postpartum oral glucose tolerance test (OGTT) was carried out at 6 weeks, and following the change in the NICE guidelines, postpartum HbA1c was carried out at 12–13 weeks following delivery. We define prediabetes as: P-FG ≥ 5. mmol/L OR P-PG ≥ 7.8 mmol/L OR P-HbA1c ≥ 40 mmol/mol ppIFG was defined as P-FG ≥ 5.6 mmol/L and ppIGT was defined as P-PG ≥ 7.8 mmol/L, respectively. We define T2DM as: P-FG ≥ 7.0 mmol/L or P-PG ≥ 11.1 mmol/L or P-HbA1c ≥ 48 mmol/mol.^26^ NGT is considered otherwise. We provide the definitions of Normalcy, Prediabetes, and Incident diabetes based on the different measures in Table 1.

**Table 1:** Definitions of Normalcy, Prediabetes, and Incident diabetes based on the different measures

Definition	Normal	Prediabetes	Incident diabetes
NICE	FPG<5.6 mmol/L (101 mg/dL) OR 2hPG<7.8 mmol/L (141 mg/dL) OR HbA1c < 40 mmol/L	FPG 5.6–6.9 mmol/L (101–126 mg/dL) OR 2hPG 7.8–11.1 mmol/L (141–200 mg/dL) OR HbA1c 40 mmol/mol.	FPG≥7.0 mmol/L (126 mg/dL) OR 2hPG≥11.1 mmol/L (200 mg/dL) OR HbA1c ≥ 6.5% (48 mmol/mol)
WHO	FPG<6.1 mmol/L (110 mg/dL) OR 2hPG<7.8 mmol/L (141 mg/dL)	FPG 6.1–6.9 mmol/L (110–125 mg/dL) OR 2hPG 7.8–11.0 mmol/L (141–198 mg/dL)	Same as NICE
ADA	FPG<5.6 mmol/L (101 mg/dL) OR 2hPG<7.8 mmol/L (141 mg/dL) OR HbA1c<5.7% (39 mmol/mol)	FPG 5.6–6.9 mmol/L (101–125 mg/dL) OR 2hPG 7.8–11.0 mmol/L (141–198 mg/dL) OR HbA1c 5.7%–6.4% (39–47 mmol/mol)	Same as NICE

### Statistical power analysis

We did a power analysis to determine if the available sample size was sufficient to identify the difference in effect between the normal and prediabetes-diagnosed GDM women. We used the *statsmodels* library and the *TTestInd-Power* class in Python to calculate the power analysis for Student’s *t* test for independent samples. For a statistical power of 90%, a minimum sample size of 130 (99 normal and 31 prediabetes) is required for the observed effect size calculated using Cohen’s *d* statistic. We provide the details of power analysis in the supplementary material.

### Machine learning

We perform machine learning (ML) in Python version 3.7. We compare LR with tree-based methods to build the prognostic model for the prediction of early prediabetes in GDM women. These algorithms inherently address the imbalance in the representation for each of the binary classes of prediabetes outcome using the ‘balanced’ parameter. The ‘balanced’ mode uses the values of *y* to automatically adjust weights inversely proportional to class frequencies in the input data, as the ratio of the total number of samples to the product of the number of classes and the number of occurrences in each class. Mathematically, the class weight is calculated as 1/(2 × fraction of women in the class). We build the tree-based model using a simple decision tree algorithm, whose performance improves using ensemble methods such as bagging and boosting. All these algorithms use hyperparameters that can significantly affect the performance of these methods on an unseen set. We determine the optimal values of these hyperparameters using nested cross-validation. More specifically, we make the entire data undergo leave-one-out cross-validation (CV1) for model evaluation and we perform an internal stratified 4-fold cross-validation (CV2) on the training folds of CV1 for hyperparameter optimization. We impute the missing values with the Multivariate Imputation by Chained Equations (MICE) technique, using the other non-missing covariates. We scale the training data in CV1 using the *StandardScaler* function and use the *saga* solver in the LR model. The *saga* solver is a variant of the stochastic average gradient (*sag*) solver that also supports the non-smooth L1 penalty, which promotes feature selection. The tree-based algorithms perform feature selection inherently, governed by the optimized hyperparameters in CV2. We perform hyperparameter optimization and model training only on the training folds (*n* − 1 samples) in CV1, with an independent set (1 sample) exclusively held out for testing. We aggregate the model predictions on each held-out sample across the *n* training folds of CV1 and plot the Receiver Operating Characteristic (ROC) curve for this aggregated set. We use the area under the ROC curve as a measure of performance. Finally, we apply it in a similar fashion on the full data to obtain the final model for deployment (Figure S3). We provide the details of the different tree-based methods employed in the supplementary materials.

### Composite risk score calculation

Using the coefficients from the final fitted LR model on the full data, we develop a composite risk scoring system using the best selected antenatal variables to predict the probability of prediabetes in GDM-diagnosed women. We calculate the composite risk score as the probability of class 1 obtained from the LR model. It is given by the expression 1/(1 + *e*^−*b*^), where *b* = *b*_0_ + *b*_1_ · *x*_1_ + *b*_2_ · *x*_2_ + *…* + *b*_*m*_ · *x*_*m*_ where *b*_0_ is the intercept and *b*_*m*_ coefficient of mth variable (*x*_*m*_), respectively.

We compute specificity, positive predictive value (PPV), negative predictive value (NPV), accuracy, and the F1 score at five predetermined values of sensitivity (60%, 70%, 75%, 80%, and 90%) for the optimal selected model. We give the definition/formulae for all these in the supplementary information section.

### Kullback-Leibler (K-L) divergence and information graphs to evaluate and compare diagnostic tests and select optimal cut-point

We use the information theory approach in Lee et al.,^27^ Samawi et al.,^28^ and Benish et al.,^29^ briefly summarized below, to select the optimal probability threshold for accurate prediction of the binary outcome of prediabetes. An important approach followed in medical diagnostics is to predict the ‘rule-in and rule-out’ potential of the diagnostic test to safely include the patients in need of treatment and discard those not in need, respectively. At a probability threshold *c* reported by the ML algorithm, suppose the proportion of the diseased population correctly predicted as diseased is given by *g*_1_(*c*) and that of the non-diseased population correctly predicted as non-diseased is given by *g*_2_(*c*). Both *g*_1_(*c*) and *g*_2_(*c*) are Bernoulli probability distributions and are simply the sensitivity and specificity, respectively at the threshold value of *c*. The K-L divergence (or relative entropy) measures the separation between these two probability distributions and is given by:

(Equation 1) $$D\left(g_{1}\|g_{2}\right)=g_{1}\left(c\right)\times \ln\frac{g_{1}\left(c\right)}{1-g_{2}\left(c\right)}+\left(1-g_{1}\left(c\right)\right)\times \ln\frac{1-g_{1}\left(c\right)}{g_{2}\left(c\right)}$$

(Equation 2) $$D\left(g_{2}\|g_{1}\right)=(1-g_{2}\left(c\right))\times \ln\frac{1-g_{2}\left(c\right)}{g_{1}\left(c\right)}+g_{2}\left(c\right)\times \ln\frac{g_{2}\left(c\right)}{1-g_{1}\left(c\right)}$$

By definition, *D*(*g*_1_∥*g*_2_) ≥ 0, *D*(*g*_2_∥*g*_1_) ≥ 0. The KL divergence is close to 0 when there is little difference between the two distributions. A high *D*(*g*_1_∥*g*_2_) value indicates the increase in information of predicting disease onset. We calculate *D*(*g*_1_∥*g*_2_) and *D*(*g*_2_∥*g*_1_) for 1000 cut points at an interval of 0.001 from 0 to 1. We chose *T*_in_ with cut-point *c*_in_ corresponding to *D*_max_(*g*_1_∥*g*_2_) as the diagnostic test with greatest rule-in potential. We chose *T*_out_ with cut-point *c*_out_ corresponding to *D*_max_(*g*_2_∥*g*_1_) as the diagnostic test with greatest rule-out potential. We calculate *P*_in_ = *e*^*D*(*g*1∥*g*2)^, which is the ratio of post-test odds to the pre-test odds of having the disease for a randomly selected diseased individual. We also calculate *P*_out_ = *e*^(*D*(*g*2∥*g*1)^, which is the ratio of pre-test odds to the post-test odds of having the disease for a randomly selected non-diseased individual. *P*_in_*, P*_out_ ≥ 1.

Next, we calculate the Information Distinguishability measure, *ID*(*g*_1_∥*g*_2_) = 1 − *e*^−*D*(*g*1∥*g*2)^ and *ID*(*g*_2_∥*g*_1_) = 1 − *e*^−*D*(*g*2∥*g*1)^, to study and compare the separation provided by the diagnostic test between the diseased and the non-diseased distributions. We calculate the objective function *TKL*_discrete_(*c*) = *D*(*g*_1_∥*g*_2_) + *D*(*g*_2_∥*g*_1_) and chose the optimal cut-point *c*_in−out_ corresponding to max(*T KL*_discrete_(*c*)) to achieve maximum information for *T*_in−out_ with high potential in both rule-in and rule-out situations. Further, we plot information graphs to characterize and compare the performance of our diagnostic tests at different cut-points depending upon the rule-in or rule-out potential. The expected value of the relative entropy provides a measure of the expected diagnostic information and plotting it as a function of the pre-test probabilities yields an information graph. The equations used to plot the information graphs are given as follows: Let *D*_i_ be the true status and *T*_i_ be the diagnostic test result for the patient, respectively, (*i* = {0, 1}, 0: disease absent, & 1: disease present). If *x* = *Pr*(*D*_1_), then the diagnostic information obtained from a +ve, and -ve test result (*I*_+_(*x*), *I*_−_(*x*), respectively) and the expected diagnostic information (IE(x)) are given as follows.

(Equation 3) $$I_{+}\left(x\right)=\frac{x\times g_{1}\left(c\right)\times \ln\left(g_{1}\left(c\right)\right)}{\mathrm{Pr}\left(T_{1}\right)}+\frac{\left(1-x\right)\times \left(1-g_{2}\left(c\right)\right)\times \ln\left(1-g_{2}\left(c\right)\right)}{\mathrm{Pr}\left(T_{1}\right)}-\ln\left(\mathrm{Pr}\left(T_{1}\right)\right)$$

(Equation 4) $$I_{-}\left(x\right)=\frac{x\times \left(1-g_{1}\left(c\right)\right)\times \ln\left(1-g_{1}\left(c\right)\right)}{1-\mathrm{Pr}\left(T_{1}\right)}+\frac{\left(1-x\right)\times g_{2}\left(c\right)\times \ln\left(g_{2}\left(c\right)\right)}{1-\mathrm{Pr}\left(T_{1}\right)}-\ln\left(1-\mathrm{Pr}\left(T_{1}\right)\right)$$

(Equation 5) $$\begin{array}{l} I_{E}\left(x\right)=x\times g_{1}\left(c\right)\times \ln\left(g_{1}\left(c\right)\right)+\left(1-x\right)\times \left(1-g_{2}\left(c\right)\right)\times \ln\left(1-g_{2}\left(c\right)\right)+\\ x\times \left(1-g_{1}\left(c\right)\right)\times \ln\left(1-g_{1}\left(c\right)\right)+\left(1-x\right)\times g_{2}\left(c\right)\times \ln\left(g_{2}\left(c\right)\right)-\\ \mathrm{Pr}\left(T_{1}\right)\times \ln\left(\mathrm{Pr}\left(T_{1}\right)\right)-\left(1-\mathrm{Pr}\left(T_{1}\right)\right)\times \ln\left(1-\mathrm{Pr}\left(T_{1}\right)\right) \end{array}$$

(Equation 6) $$\mathrm{Pr}\left(T_{1}\right)=x\times \mathrm{Pr}\left(T_{1}|D_{1}\right)+\left(1-x\right)\times \mathrm{Pr}\left(T_{1}|D_{2}\right)$$

(Equation 7) $$=x\times g_{1}\left(c\right)+\left(1-x\right)\times \left(1-g_{2}\left(c\right)\right)$$

In addition, we also plot the information graph by representing the total K-L divergence as the discrete Bregman divergence, which is the sum of the vertical distances between the negative Shannon entropy function (see supplementary material for details) and tangents to it at probabilities *p* = *g*_1_(*c*) and p = 1 − *g*_2_(*c*).

### Decision curve analysis

We carry out decision curve analysis (DCA) to evaluate and compare the performance of our model in comparison to the ‘treat all’ and ‘treat none’ approaches. Finally, we compare the correctly identified non-attenders (sensitivity) vs. follow-ups avoided (the true negatives + false negatives, obtained from the optimal selected model), to calculate the number of women requiring enhanced care, to maximize targeted postpartum follow-up.

## Results

**Figure 1:**
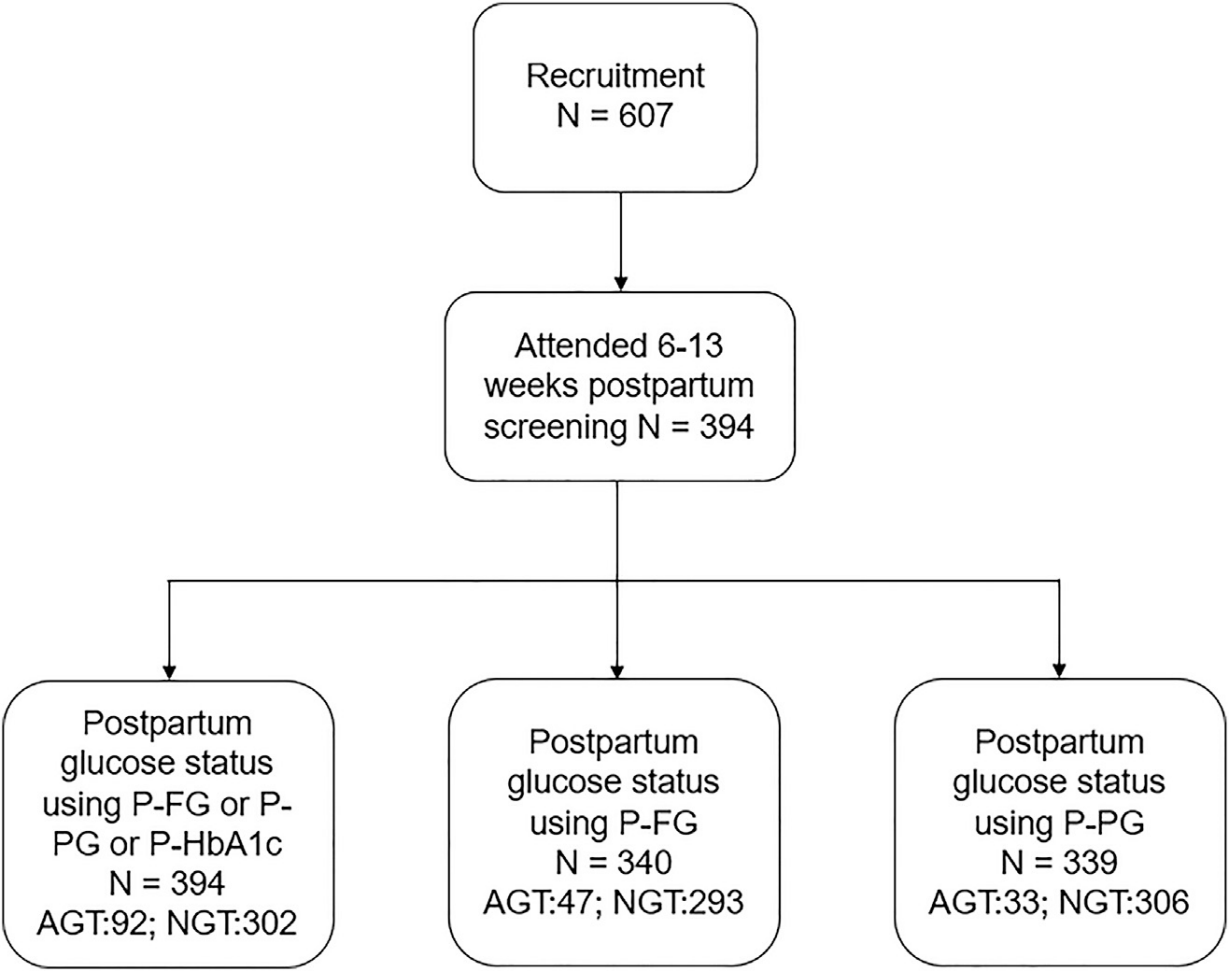
Consort diagram of early postpartum glucose tolerance The flow chart displays the proportion of GDM women with and without prediabetes. The diagnosis of prediabetes was made if: FPG ≥5.6 or 2-h glucose ≥7.8 at postpartum OGTT or HbA1c ≥ 40 mmol/mol.

Postpartum glucose status was available for 394 (64.91%) out of the 607 women (Figure 1). 340 (56.01%) women underwent OGTT at 6 weeks and 128 (21.09%) underwent the postpartum HbA1c around 13 weeks prediabetes is present in 92 (23.35%) women. Of these 47 (51.09%) were abnormal by P-FG, 33 (35.87%) by P-PG, and 39 (42.39%) by P-HbA1c. We show the baseline characteristics of these 394 women in Table 2.

**Table 2:** Comparison of antenatal, delivery and postnatal characteristics of GDM women with presence and absence of prediabetes

Variable	All attended N = 394	Prediabetes N = 92	ppNGT N = 302	Missing ppGT N = 213
Maternal characteristics				
Age	32.21 ± 5.40	32.38 ± 5.46	32.16 ± 5.39	30.45 ± 6.22
Height (m)	1.64 ± 0.07	1.64 ± 0.07	1.64 ± 0.07	1.64 ± 0.07
Weight (kg)	79.78 ± 19.80	84.32 ± 22.82	78.36 ± 18.58	85.58 ± 21.27
BMI (kg/mˆ2)	29.76 ± 6.81	31.21 ± 7.40	29.30 ± 6.56	31.79 ± 7.54
Systolic BP (mmHg)	115.71 ± 13.62	116.07 ± 13.78	115.60 ± 13.59	115.97 ± 12.54
Diastolic BP (mmHg)	69.98 ± 9.40	70.41 ± 8.18	69.85 ± 9.76	70.74 ± 9.57
Parity				
1	192 (48.98%)	43 (46.74%)	149 (49.67%)	73 (34.27%)
≥2	200 (51.02%)	49 (53.26%)	151 (50.33%)	139 (65.26%)
Ethnicity				
White European	303 (76.90%)	66 (71.74%)	237 (78.48%)	178 (83.57%)
South Asian	46 (11.68%)	13 (14.13%)	33 (10.93%)	21 (9.86%)
Others	45 (11.42%)	13 (14.13)	32 (10.60%)	14 (6.57%)
Smoking category				
Never smoked	190 (50.94%)	43 (49.43%)	147 (51.40%)	80 (37.56%)
Ex-smoker	147 (39.41%)	34 (39.08%)	113 (39.51%)	69 (32.39%)
Smoker	36 (9.65%)	10 (11.49%)	26 (9.09%)	61 (28.64%)
Marrital Status				
Single	21 (5.74%)	3 (3.45%)	18 (6.45%)	26 (12.21%)
Employment own/partner				
Unemployed	9 (2.56%)	3 (3.53%)	6 (2.25%)	9 (4.23%)
At OGTT and Intrapartum				
GA at antenatal OGTT (weeks)	28.16 ± 4.21	27.50 ± 4.08	28.37 ± 4.23	27.53 ± 4.74
A-FG (mmol/L)	4.95 ± 0.87	5.38 ± 0.91	4.82 ± 0.81	5.09 ± 0.81
A-PG (mmol/L)	8.55 ± 1.75	8.90 ± 1.75	8.44 ± 1.74	8.13 ± 1.75
A-HbA1c (mmol/mol)	35.52 ± 4.69	38.13 ± 4.61	34.72 ± 4.42	36.17 ± 5.70
GA birth (weeks)	37.91 ± 1.27	37.65 ± 1.28	37.99 ± 1.26	37.95 ± 1.40
Preterm (GA ≤ 37 weeks)	53 (13.59%)	18 (20.00%)	35 (11.67%)	32 (15.02%)
Delivery mode				
Spontaneous	197 (50.38%)	37 (40.66%)	160 (53.33%)	112 (52.58%)
Instrument assisted	32 (8.18%)	6 (6.59%)	26 (8.67%)	18 (8.45%)
Caesarean delivery	162 (41.43%)	48 (52.75%)	114 (38.00%)	79 (37.09%)
Neonatal characteristics				
Birth weight (grams)	3211.95 ± 467.75	3216.48 ± 511.41	3210.57 ± 454.58	3201.72 ± 531.36
Birth Centile				
AGA (10-90th centile)	267 (74.58%)	61 (72.62%)	206 (75.18%)	133 (62.44%)
SGA (<10 centile)	42 (11.73%)	10 (11.90%)	32 (11.68%)	38 (17.84%)
LGA (>90 centile)	49 (13.69%)	13 (15.48)	36 (13.14%)	27 (12.68%)
Male baby	183 (46.80%)	42 (46.15%)	141 (47.00%)	124 (58.22%)
Breastfeeding initiated	207 (58.31%)	45 (54.88%)	162 (59.34%)	86 (40.38%)
Postpartum maternal biochemical characteristics				
P-FG (mmol/L)	4.99 ± 0.62	5.64 ± 0.79	4.78 ± 0.38	–
P-PG (mmol/L)	5.59 ± 1.62	7.10 ± 2.08	5.12 ± 1.07	–
P-HbA1c (mmol/mol)	37.53 ± 4.84	42.22 ± 4.56	34.99 ± 2.55	–

### Machine learning analysis

**Figure 2:**
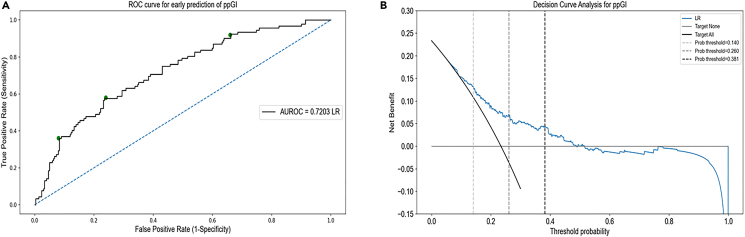
Estimated ROC for the prediction of postpartum prediabetes following a GDM pregnancy (A) AUROC (Area under the receiver operating characteristic) was used to evaluate the performance of our machine learning-based method using the logistic regression model on the validation cohort, n = 394 by aggregating the predictions from the test folds of CV1. The area under ROC was 0.7203. The green dots on the ROC curve represent *T*_in_ (*c*_in_ = 0.381), *T*_in−out_ (*c*_in−out_ = 0.260), and *T*_out_ (*c*_out_ = 0.140), from left to right, respectively. (B) The decision curve analysis (DCA) showed the net benefit obtained from the ML (blue) prediction model. The net benefit of implementing our model in a clinical setting is larger when compared to the follow-up of all GDM women for prediabetes. DCA was derived from the equation, Net benefit ^TP−FP×(*p*^*t*^*/*1−*p*^*t*^)^ = _N_, where TP and FP are the true positives and false positives respectively, *p*_*t*_ is the probability threshold, and N is the total number of participants in the validation cohort, n = 607.

The data are imbalanced (as expected), with a 23.35% representation of the positive prediabetes class. We compare simple LR with different classification tree methods for predicting prediabetes from training on this small and imbalanced dataset. We use class-weight = balanced in the LR algorithm and ‘balanced’ classification tree-based algorithms from the imbalanced-learn python package for developing the tree-based prognostic models. The predictive performance of our proposed framework improves significantly by applying ensemble methods of bagging and boosting to the base decision tree estimator but remains lower than LR. LR gives the area under the ROC curve of 0.7203 from aggregating the test predictions from the leave-one-out cross-validation (Figure 2A). The Brier score loss for calibration of the LR model is 0.1530 and the calibration plot is shown in Figure S9. The mean CV-accuracy as a function of the regularization constant ’C’ is shown in Figure S5. LR gives the area under the ROC curve of 0.6598 for postpartum fasting glucose prediction (Figure S6). Using the base decision tree algorithm and leave-one-out cross-validation, the area under the ROC curve for the aggregated test predictions is 0.6210, bagging decision trees improves it to 0.6883. Random forests further improve it to 0.6944 using 4-fold stratified cross-validation in CV1 and the maximum area under the ROC curve from the tree-based algorithms is 0.6991 from balanced bagging using histogram-based gradient boosting tree classification algorithm using 4-fold stratified cross-validation (Figure S4). We use 4-fold stratified cross-validation in CV1 instead of leave-one-out for random forests and the boosting algorithm due to the high time complexity of leave-one-out. Other boosting algorithms like XGBoost, LightGBM, and CatBoost give the area under the ROC curve of 0.6427, 0.6646, and 0.6948 respectively. We conclude that the simplest prediction algorithm for binary classification, LR, outperforms the advanced tree-based methods in the prediction of prediabetes. Our final composite risk score using the LR model with A-FG and A-HbA1c is highly robust for the prediction of prediabetes in GDM women. Out of the n = 394 runs of leave-one-out cross-validation, antenatal fasting glucose and antenatal HbA1c are selected 318 (> 80%) times. The shap summary plots generated using the tree explainer package in Python provide additional evidence supporting the finding that A-FG and A-HbA1c are the sole significant predictors of postpartum prediabetes in women with GDM (Figures S7 and S8).

### Composite risk score calculation

Based on our proposed final LR model, we calculate the composite risk score, *c* (or P(prediabetes)), as,

(Equation 8) $$P\;\left(\text{prediabetes}\right)=\frac{1}{1+e^{-\left(-8.36+0.58\times A-\text{FG}+0.10\times A-\text{HbA}1c\right)}}$$

The association results of the LR model between the risk predictors and pre-diabetes outcome are given in Table 3.

**Table 3:** Factors associated with postpartum prediabetes by machine learning model

Variables	Β (SE)	OR (95% CI)	p value
A-FG (mmol/L)	0.5816 (0.207)	1.79 (0.175, 0.988)	0.005
A-HbA1c (mmol/mol)	0.0996 (0.038)	1.11 (0.025, 0.174)	0.009

### Kullback–Leibler (K-L) divergence and information graphs to evaluate and compare diagnostic tests and select optimal cut-point

*T*_in_ with *D*_max_(*g*_1_(*c*)*, g*_2_(*c*)) = 0.30 and *c*_in_ = 0.381 has high specificity of 92%, in concurrence with the ‘rule-in-specific-test’ principle and *T*_out_ with *D*_max_(*g*_2_(*c*)*, g*_1_(*c*)) = 0.28 and *c*_out_ = 0.140 has high sensitivity of 92%, again in concurrence with the ‘r-out-sensitive-test’ principle. *P*_in_ = 1.35 and *P*_out_ = 1.23 for *T*_in_, and *P*_in_ = 1.21 and *P*_out_ = 1.33 for *T*_out_, which is the increase (decrease) in disease odds after the test for a diseased (control) individual. *T*_in−out_ with max(*T KL*_discrete_(*c*)) = 0.51 for *c*_in−out_ = 0.260 has *P*_in_ = 1.31 and *P*_out_ = 1.27. Also, maximum of the Youden’s index, *J*_max_ = 0.34 (*J*(*c*) = *g*_1_(*c*) + *g*_2_(*c*) − 1), and maximum *F*_1_-score = 0.49 occurs at the same *c*_in−out_ = 0.260. *e*^(*T*in(*KL*in)−*T*out(*KL*in))^ = *e*^(0.30−0.19)^ = 1.12 > 1, which implies that positive result obtained by *T*_in_ is more likely to be true than positive result obtained by *T*_out_. In other words, *T*_in_ is more specific and yields fewer false positives compared to *T*_out_. Similarly, *e*^(*T*in(*KL*out)−*T*out(*KL*out))^ = *e*^(0.21−0.28)^ = 0.93 < 1 shows that *T*_in_ is less sensitive with more false negatives.

**Figure 3:**
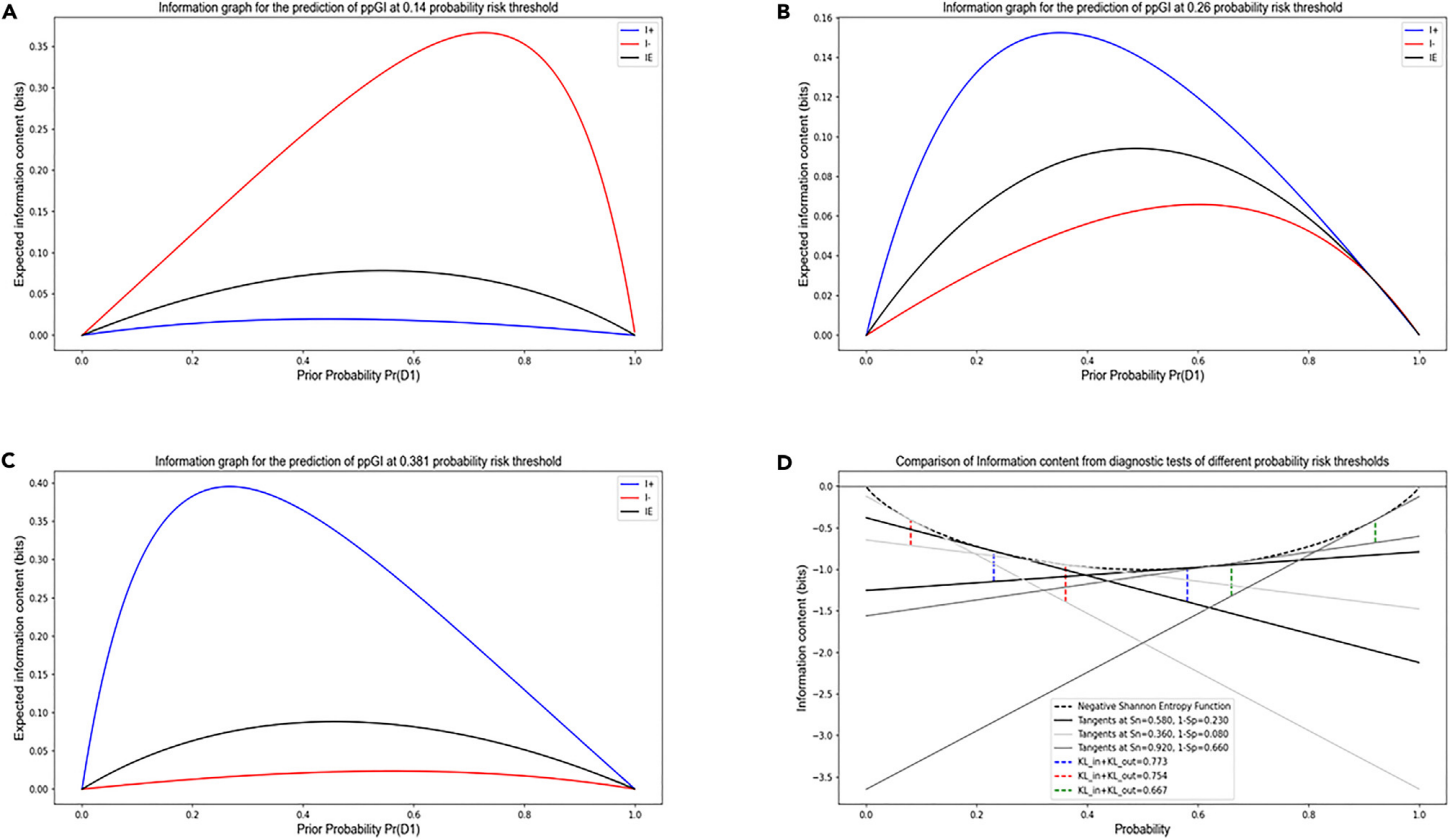
Information graphs for comparing rule-in and rule-out test potentials for predicting a low and high risk of prediabetes post-GDM Information graphs provide means to distinguish between diagnostic test performance. We compared the diagnostic information obtained from *T*_out_, *T*_in−out_, and *T*_in_ defined by the cut-points 0.140, 0.260, 0.381. A positive diagnosis made by the ‘rule-in-specific-test’ and a negative diagnosis made by the ‘rule-out-sensitive-test’ gives us the most information, as expected. (A–C) Maximum information from a positive test diagnosis (blue) is obtained at a lower pre-test probability than the maximum information from a negative test diagnosis (red). The diagnostic test with a lower cut-point gives maximum information when the diagnosis is negative (i.e., the test is very sensitive and we can rule out the negative cases safely) and the diagnostic test with a higher cut-point gives maximum information when the diagnosis is positive (i.e., the test is very specific to the disease and we can rule in the positive cases safely). *I*_E_ is the expected information from the diagnostic test (*x* × *I*_+_ + (1 − *x*) × *I*_−_, where *x* is the probability of a positive test diagnosis). (D) The sum of the distances between the tangents to the negative Shannon entropy function at *p* = *g*_1_(*c*) and p = 1 − *g*_2_(*c*) is the discrete Bregman divergence, which represents total K-L divergence.

We generated the information graphs using the equations for *I*_+_(x), *I*_−_(x), and *I*_E_(x) as a function of *x* = *Pr*(*D*_1_), as shown in Figures 3A–3C. We can observe that *T*_in_ provides the most diagnostic information when the test result is positive, and the pre-test probability of a positive result (*Pr*(*D*_1_)) is low. *T*_out_ provides the most diagnostic information when the test result is negative, and the pre-test probability of a positive result is high. For *T*_in−out_, we obtain more diagnostic information when the test yields a positive result than a negative one and we obtain maximum information from a positive result at a lower pre-test probability than that from the negative result. In Figure 3D, we can see the information gained using the discrete Bregman divergence representation of *TKL*_discrete_ by adding the vertical distances from the negative Shannon Entropy function to the tangents drawn at probability *p* = *g*_1_(*c*) and 1 − *g*_2_(*c*).

Using the prognostic model with LR, 15 out of 100 women are above the optimal threshold of 0.381, and focusing on these women could improve the early prediabetes diagnosis. 28 out of 100 women are below the optimal threshold of 0.140, and testing for early prediabetes diagnosis can be safely avoided in this category. The model shows 92% sensitivity for the rule-in test and 92% specificity for the rule-out test, Table 4 shows the sensitivity, specificity, PPV, NPV, F1 score, accuracy, and other measures related to K-L divergence at different probability thresholds.

**Table 4:** Performance of the diagnostic test for postpartum prediabetes at various probability thresholds

Algo	c	g_1_(c)	g_2_(c)	PPV	NPV	F1	Accu	J	KL_in_	KL_out_	TKL	P_in_	P_out_	ID_in_	ID_out_	TA
LR	0.145	0.9	0.36	0.3007	0.9237	0.4511	0.4873	0.2631	0.1833	0.2508	0.4342	1.2012	1.2851	0.1675	0.2219	30
	0.169	0.8	0.48	0.3217	0.8902	0.4596	0.5584	0.2878	0.1792	0.2086	0.3878	1.1963	1.2319	0.1641	0.1883	42
	0.193	0.75	0.57	0.3467	0.8821	0.4742	0.6117	0.3195	0.2106	0.2299	0.4405	1.2344	1.2585	0.1899	0.2054	49
	0.201	0.7	0.62	0.3556	0.8692	0.4706	0.6345	0.3115	0.1986	0.206	0.4046	1.2197	1.2288	0.1801	0.1862	54
	0.239	0.62	0.71	0.3904	0.8589	0.479	0.6853	0.3249	0.2255	0.2164	0.4419	1.253	1.2416	0.2019	0.1946	63
T_out_	0.14	0.92	0.34	0.2982	0.9358	0.4509	0.4746	0.2617	0.1942	0.2829	0.4771	1.2144	1.3269	0.1765	0.2464	28
T_in_	0.381	0.36	0.92	0.569	0.8244	0.44	0.7868	0.2759	0.2965	0.2068	0.5033	1.3451	1.2298	0.2566	0.1868	85
T_in-out_	0.26	0.58	0.76	0.4274	0.8556	0.4907	0.7208	0.341	0.2661	0.2407	0.5069	1.3049	1.2722	0.2337	0.214	69
DTC	0.113	0.64	0.52	0.1878	0.8934	0.2902	0.5406	0.1617	0.0528	0.0542	0.107	1.0542	1.0557	0.0515	0.0528	50
	0.114	0.6	0.57	0.1934	0.892	0.2929	0.5711	0.1689	0.0575	0.058	0.1154	1.0591	1.0597	0.0558	0.0563	54
T_out_	0.871	0.26	0.93	0.3947	0.8792	0.3125	0.8325	0.1902	0.1745	0.1217	0.2962	1.1906	1.1294	0.1601	0.1146	90
T_in_	0.871	0.26	0.93	0.3947	0.8792	0.3125	0.8325	0.1902	0.1745	0.1217	0.2962	1.1906	1.1294	0.1601	0.1146	90
T_in-out_	0.871	0.26	0.93	0.3947	0.8792	0.3125	0.8325	0.1902	0.1745	0.1217	0.2962	1.1906	1.1294	0.1601	0.1146	90
Bagging DTC	0.244	0.9	0.21	0.164	0.9221	0.2773	0.3122	0.1079	0.041	0.0498	0.0909	1.0419	1.0511	0.0402	0.0486	20
	0.306	0.81	0.32	0.1703	0.9068	0.2814	0.3909	0.1288	0.042	0.0471	0.089	1.0429	1.0482	0.0411	0.046	30
	0.349	0.76	0.45	0.1913	0.9146	0.3056	0.4924	0.205	0.0906	0.1001	0.1907	1.0949	1.1053	0.0866	0.0952	42
	0.37	0.71	0.51	0.1981	0.9091	0.3094	0.5355	0.2128	0.0932	0.0992	0.1925	1.0977	1.1043	0.089	0.0945	47
	0.468	0.6	0.76	0.307	0.9179	0.407	0.7411	0.3683	0.3083	0.2808	0.5891	1.3611	1.3242	0.2653	0.2449	71
T_out_	0.468	0.6	0.76	0.307	0.9179	0.407	0.7411	0.3683	0.3083	0.2808	0.5891	1.3611	1.3242	0.2653	0.2449	71
T_in_	0.564	0.45	0.89	0.4194	0.9036	0.4333	0.8274	0.3411	0.376	0.2765	0.6525	1.4565	1.3184	0.3134	0.2415	84
T_in-out_	0.564	0.45	0.89	0.4194	0.9036	0.4333	0.8274	0.3411	0.376	0.2765	0.6525	1.4565	1.3184	0.3134	0.2415	84
RFC	0.371	0.9	0.29	0.1793	0.9423	0.2989	0.3807	0.1882	0.104	0.1354	0.2394	1.1096	1.145	0.0988	0.1266	26
	0.431	0.81	0.47	0.208	0.9345	0.331	0.5178	0.2776	0.1689	0.1979	0.3667	1.184	1.2188	0.1554	0.1795	43
	0.446	0.76	0.5	0.2085	0.9235	0.3271	0.5406	0.2616	0.1436	0.1591	0.3027	1.1544	1.1725	0.1337	0.1471	46
	0.466	0.71	0.56	0.2158	0.9167	0.3306	0.5787	0.2634	0.1417	0.1501	0.2918	1.1522	1.1619	0.1321	0.1394	52
	0.502	0.6	0.66	0.2365	0.9065	0.3398	0.6548	0.2671	0.1486	0.1452	0.2938	1.1602	1.1563	0.1381	0.1351	62
T_out_	0.618	0.4	0.89	0.3833	0.8952	0.3898	0.8173	0.2864	0.2737	0.2046	0.4782	1.3148	1.227	0.2394	0.185	85
T_in_	0.729	0.17	0.99	0.6667	0.8734	0.274	0.8655	0.1575	0.2782	0.1352	0.4134	1.3207	1.1448	0.2428	0.1265	96
T_in-out_	0.618	0.4	0.89	0.3833	0.8952	0.3898	0.8173	0.2864	0.2737	0.2046	0.4782	1.3148	1.227	0.2394	0.185	85
Bagging HGBC	0.173	0.9	0.24	0.1699	0.9318	0.2857	0.3401	0.1406	0.0641	0.0805	0.1447	1.0662	1.0838	0.0621	0.0774	22
	0.226	0.81	0.37	0.1808	0.9179	0.2956	0.4315	0.1764	0.0742	0.0851	0.1593	1.0771	1.0888	0.0715	0.0816	34
	0.274	0.76	0.45	0.1921	0.9152	0.3066	0.4949	0.208	0.0931	0.1029	0.196	1.0976	1.1083	0.0889	0.0977	42
	0.308	0.74	0.5	0.2028	0.9176	0.3185	0.533	0.2384	0.1187	0.1296	0.2482	1.126	1.1383	0.1119	0.1215	46
	0.366	0.71	0.59	0.2303	0.9213	0.3475	0.6091	0.2992	0.1828	0.1922	0.375	1.2006	1.212	0.1671	0.1749	55
T_out_	0.562	0.47	0.87	0.375	0.9037	0.4154	0.8071	0.3316	0.322	0.2512	0.5732	1.3799	1.2855	0.2753	0.2221	82
T_in_	0.562	0.47	0.87	0.375	0.9037	0.4154	0.8071	0.3316	0.322	0.2512	0.5732	1.3799	1.2855	0.2753	0.2221	82
T_in-out_	0.562	0.47	0.87	0.375	0.9037	0.4154	0.8071	0.3316	0.322	0.2512	0.5732	1.3799	1.2855	0.2753	0.2221	82

### Decision curve analysis

In the decision curve analysis by comparing the ‘treat all’ and ‘treat none’ approaches, the ML model obtains a higher standardized net benefit as compared to the universal screening of all GDM women for early prediabetes (Figure 2B).

## Discussion

In this study, we try to predict at the time of delivery if the women diagnosed with GDM are at high risk of getting diagnosed with postpartum prediabetes at 6–13 weeks postpartum. For this purpose, we employ a variety of machine learning techniques including both LR and advanced tree-based algorithms and train the models using routinely collected antenatal and delivery variables as predictors. Our proposed model using nested cross-validation and LR algorithm can effectively predict prediabetes in GDM women, using only the antenatal predictors fasting glucose and HbA1c, with good sensitivity and specificity. The proposed model has the capability to serve as a valuable tool for prediction and targeted screening for postpartum prediabetes in women with GDM during the antenatal period itself. By identifying individuals at higher risk, healthcare providers can implement timely interventions to target postpartum weight retention, which has shown to be an independent predictor of future prediabetes/diabetes, through personalized lifestyle modifications. This proactive approach can help to prevent or delay the onset of type 2 diabetes, improve long-term health outcomes, and reduce healthcare costs associated with managing diabetes-related complications.

The use of machine learning for predicting postpartum prediabetes in GDM-diagnosed women has been rarely studied. We are aware of only two studies that have made use of machine learning algorithms to predict the occurrence of T2DM post-GDM: Kumar et al.^19^ and Krishnan et al.^30^ Krishnan et al. proposed random forest and Gaussian naive Bayes algorithms to predict T2DM after GDM, and achieved a modest specificity of 23% at a sensitivity of 88%. It also lacked the use of advanced techniques to deal with imbalanced data. Real-world medical data are scarce due to the different challenges posed in its collection. To the best of our knowledge, there is no larger data collected for studying prediabetes in GDM women than the data in the present study. In our study, we propose a more personalized approach to identifying postpartum prediabetes after GDM, at the antenatal visit itself, by calculating a simple score based on only two easy-to-measure biochemical predictors, obtained using machine learning techniques and a LR algorithm, with good sensitivity and specificity (each of 92% for rule-out and rule-in tests, respectively). Further, we suggest different cut-offs for classifying high-risk women depending upon resource availability.

The proposed prediction test needs only the antenatal fasting glucose (at the time of antenatal OGTT) and HbA1c, usually measured soon after the diagnosis of GDM for clinical use. Thus, no additional tests/costs are involved, and is easy to use by healthcare professionals. The information theory analysis proposes different cut-offs for classification according to the requirement of ruling-in or ruling-out the prediabetes condition in GDM-diagnosed women. All women diagnosed with GDM during pregnancy are recommended to have annual screening,^25^^,^^31^ although the compliance is currently poor.^5^^,^^24^ Therefore, we can allow for more false positives than false negatives and propose *c*_out_ = 0.140 as the optimal cut-off for classification. However, in low-resource settings, we can primarily focus on women with *P* (prediabetes) ≥ *c*_in_ = 0.381 and then consider women with *P* (prediabetes) ≥ *c*_in−out_ = 0.260 in the following step. If resource constraint is not an issue, we can target women with *P* (prediabetes) ≥ *c*_out_ = 0.140 as well. Targeting GDM women stepwise according to their risk of developing prediabetes is more personalized than the blanket approach of targeting all women with GDM. This could be a pragmatic approach in settings with limited resources. The desired cut-off out of *c*_in_, *c*_out_, or *c*_in−out_ can be chosen depending upon the purpose and setting in which this diagnostic test is used.

Postpartum weight loss has been shown to reduce the risk of incidence of T2DM and recurrent GDM in the subsequent pregnancy.^32^^,^^33^ However, initiating such lifestyle interventions can be difficult due to lack of personalization and may not produce optimum results due to poor adherence by the women.^34^ Our approach to identifying women with a high risk of prediabetes (using any ‘*c*’) can provide an improved understanding of individualized prediabetes risk which can be used to target women for interventions (diet and lifestyle, encourage breastfeeding, etc) for postpartum weight loss. This can in turn improve their T2DM and CVD risk profile. Women are most conducive to interventions during pregnancy and also maintain close contact with healthcare professionals. Identifying the high-risk women during the antenatal visits will help the healthcare professionals to implement necessary interventions throughout the remaining pregnancy period, and also encourage postpartum follow-up. These strategies can include personalized monitoring, education and support on lifestyle changes and early treatment, if necessary, for high risk women. Inexpensive medications such as metformin have been shown to prevent type 2 diabetes in women with a history of GDM and may provide added benefit in high risk women. In addition, empowering high risk women with knowledge about healthy lifestyle choices, self-care practices, and potential risk factors can facilitate informed decision-making and sustained behavior change.

We believe that the results obtained are supportive for testing and validating our rule-in and rule-out composite risk score approach on a larger prospective dataset. Also, real-world validation of machine learning models is an essential step in ensuring their effectiveness and reliability. Real-world validation of trained ML models requires an understanding of domain shift, continuous monitoring of model performance, data collection for recalibration, and the application of techniques like active learning, transfer learning, and domain adaptation. As and when we get access to more datasets of similar high quality from the field, the model can certainly be updated, ensuring, as in this paper, that there is no contamination of training data with test data during model updation. It would not be advisable to update ML models in real time on the field, because of the need to ensure data quality as well as lack of contamination in training the model.

### Strengths and limitations

The key strength of our study is the use of a variety of machine learning techniques and the comparison of the LR algorithm with tree-based algorithms for developing the prognostic model for individualized risk prediction of prediabetes following GDM pregnancy. In addition, to the best of our knowledge, this is the first study that used K-L divergence and information graphs for evaluating and comparing different diagnostic tests at different cut-points and explaining their rule-in and rule-out potentials. However, our study has important limitations. First, this is retrospective data and hence other potential variables that could influence the prediabetes status such as gestational weight gain and insulin treatment were not electronically available. Second, postpartum glucose status data were only available in 65.0% of the cohort, although this follow-up rate for postpartum glucose testing was higher than the national average. Finally, while the sample size is small (n = 394 and n = 92 for the prediabetes class) for machine learning analyses, this was adequate based on the substantial predictive performance and the power calculations. In addition, the only available literature to our knowledge that looked at predicting the onset of T2DM following GDM was based on only 77 patient records with 15 variables.^30^ Validation with future datasets will be useful, and our model opens avenues for other clinicians to expand in the future.

### Conclusions

This study shows that our proposed model using a LR algorithm is effective for the prediction of prediabetes in GDM women by using the already available antenatal fasting glucose and antenatal HbA1c. We believe that this approach is easy for practical use with no additional cost and could be extremely effective for individualized risk stratification of GDM women. This approach could be used for targeted glucose testing during the postpartum period in a resource-constrained setting.

## STAR★Methods

### Key resources table

**Table:** 

REAGENT or RESOURCE	SOURCE	IDENTIFIER
**Deposited data**		

Available upon request following the completion of a suitable confidentiality agreement.		

**Software and algorithms**		

Python version 3.7	Python Software Foundation	https://www.python.org

### Resource availability

#### Lead contact

Further information and data will be available upon request following the completion of a suitable confidentiality agreement by lead contact, P Saravanan (p.saravanan@warwick.ac.uk).

#### Materials availability

All materials are available upon request.

### Experimental model and study participation details

Not Applicable.

### Method details

Details of the tree-based algorithms.

#### Balanced decision tree

A single decision rule is developed from learning from the training data in each iteration i of CV1 and is used to make predictions on the held-out test data. The number of features used in the decision rule, their order, the split-ting cut-offs at each node in the decision tree, etc. are decided by optimizing the hyperparameters: [max leaf nodes, min samples split, min samples leaf, criterion] in CV2.

#### Balanced bagging using decision tree

When the training data is small, b different bootstrapped training datasets can be generated by sampling with replacement from the original training data. The model is trained on each of these b training datasets to get fb(x) and the final classification model is obtained by averaging all the b predictions,

(Equation 9) fbag(x)=1Bfbb=1B(x).fbag(x)

*f*_*bag*_(*x*) is used to make predictions on the held-out test data. The decision tree hyperparameters optimized are same as above.

#### Balanced random forest

Random forests are similar to bagged decision trees except for the number of features considered at each split in the decision tree-all features are split candidates in bagged decision trees vs. a random sample of m predictors are the split candidates in random forests. The hyperparameters for optimiza-tion are similar as for decision trees, except that criterion is replaced by m which is either sqrt or log2.

#### Balanced bagging using histogram-based gradient boosting tree

Boosting works in a similar fashion to bagging, however the individual decision trees are grown sequentially using information from previously grown trees, and on modified version of the original training dataset. The hyperparameters optimized in this method in CV2 are: [max leaf nodes, min samples leaf, max depth, l2 regularization]. The LR model could predict ppIFG with an area under the ROC curve of 0.6598.

### Quantification and statistical analysis

In statistics, power analysis is used to determine the probability of finding a significant difference between two sample distributions, if it exists. A statistical hypothesis test makes an assumption about the outcome. The null hypothesis in a statistical test is that there is no significant difference between specified populations, any observed difference is due to sampling or experimental error. The statistical power is the probability of correctly rejecting the null hypothesis. Therefore, in mathematical terms, power can be defined as probability of True positives (TP). For a predefined significance level and known effect size, we can either fix power and calculate minimum required sample size to obtain the desired effect or calculate power for the available sample size. Antenatal fasting (ANF) and antenatal HbA1c (ANHbA1c) are the two selected predictors for antenatal prediction of prediabetes in GDM diagnosed women. The sample distributions for ANF and ANHbA1c for the GDM (class 1) and non-GDM (class 0) groups are as shown in Figures S1A and S1B, respectively. Let r be the ratio of the number of samples in the second sample distribution to those in the first. Then r = Nobs2/Nobs1 = 92/302 = 0.305. Calculating effect size We will use the Cohen’s d for calculating the effect size. Let *η*_1_, and *η*_2_ be the number of samples in distribution 1 (class 0) and distribution 2 (class 1), respectively. Let *μ*_1_, and *μ*_2_ be the means and *σ*_1_, and *σ*_2_ be the standard deviations of the two sample distributions. Then, the Cohen’s d statistic is given by (Cohen, Jacob. Statistical power analysis for the behavioral sciences. Academic press, 2013.):

(Equation 10) $$d=\frac{\mu_{1}-\mu_{2}}{\left(\frac{\sqrt{\left(\eta_{1}-1\right)\cdot \sigma_{1}^{2}+\left(\eta_{2}-1\right)\cdot \sigma^{2}}}{\eta_{1}+\eta_{2}-2}\right)}$$

Assuming the sample distributions of ANF and ANHbA1c for class 0 and class 1 are normal, we get dANF = 0.681 and dHbA1c = 0.781. Calculating Sample size for fixed Power Let us fix significance level = 0.05 and statistical power p = 0.9. Using the Cohen’s d calculated above, we get the minimum required sample size as 130 (99 class 0 + 31 class 1) for ANF and 99 (76 class 0 + 23 class 1) for ANHbA1c. Lastly, we plotted power curves to see how the power of the test changes with the other parameters: sample size, effect size, and significance level. In Figure S2, we can see how the power of the test increases with increasing sample size, for different fixed effect sizes. We can understand that if the effect size is small (greater overlap between the two sample distributions), then greater number of observations are required to identify the existing significant difference between the two sample distributions, and thus correctly reject the null hypothesis. Also, the power of the test increases with increasing effect size. Basic formulae F1 score: 2 × Precision × Recall/(Precision + Recall) Negative Shannon entropy function: h(p) = p × ln (p) + (1-p) × ln (1-p).

## Data Availability

The full dataset is available upon request following the completion of a suitable confidentiality agreement.
