# Supplementary material for: Prediction of postpartum prediabetes by machine learning methods in women with gestational diabetes mellitus
Source: iScience. 2023 Sep 9;26(10):107846. doi: 10.1016/j.isci.2023.107846 (PMC10520542; doi:10.1016/j.isci.2023.107846)
Supplement: Document S1. Figures S1–S10 [file mmc1.pdf]

iScience, Volume *26*

Supplemental information

Prediction of postpartum prediabetes by machine learning methods in women with gestational diabetes mellitus

Durga Parkhi, Nishanthi Periyathambi, Yonas Ghebremichael-Weldeselassie, Vinod Patel, Nithya Sukumar, Rahul Siddharthan, Leelavati Narlikar, and Ponnusamy Saravanan

<u>Supplementary Material</u>

Fig S1: Sample distributions of the features selected by the prediction model, related to Figure 1.

(a) The sample distributions for ANF (b) The sample distributions for ANHbA1c

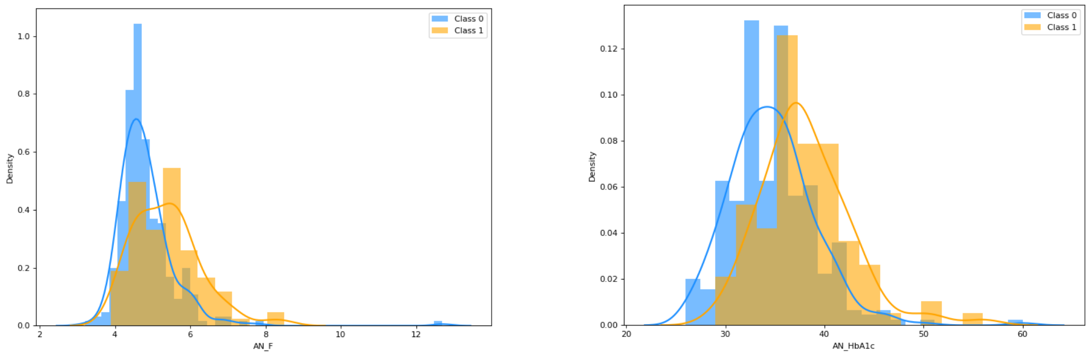

Fig S2: Change in power of the test as a function of sample size, for fixed effect sizes, related to Figure 1.

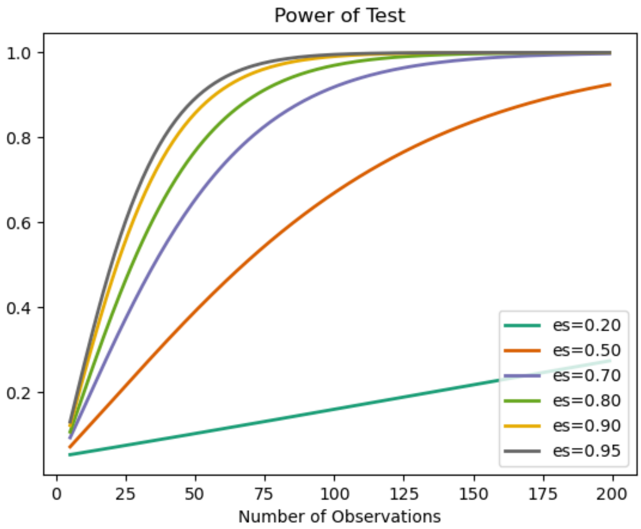

Fig S3: Concept diagram of our proposed model. Replace leave-one-out with 4-fold stratified cross validation for the model using Random forests and boosting algorithms, related to Figure 2.

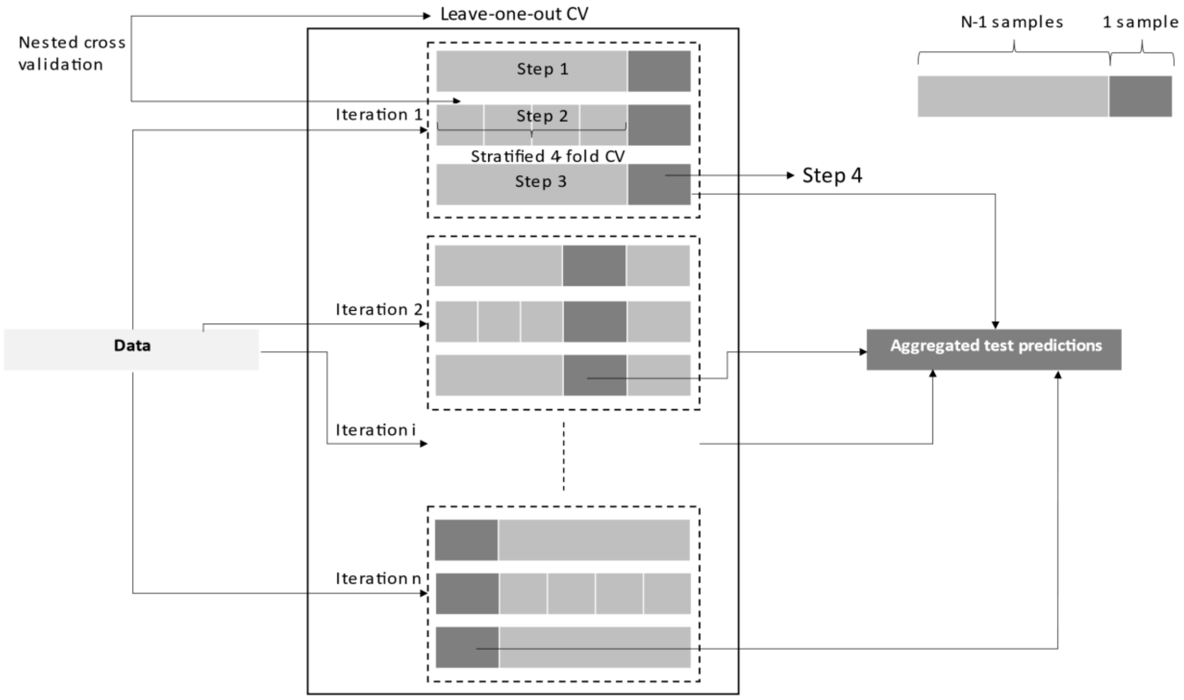

Fig S4: ROC curve for early prediabetes prediction using tree-based methods, related to Figure 2.

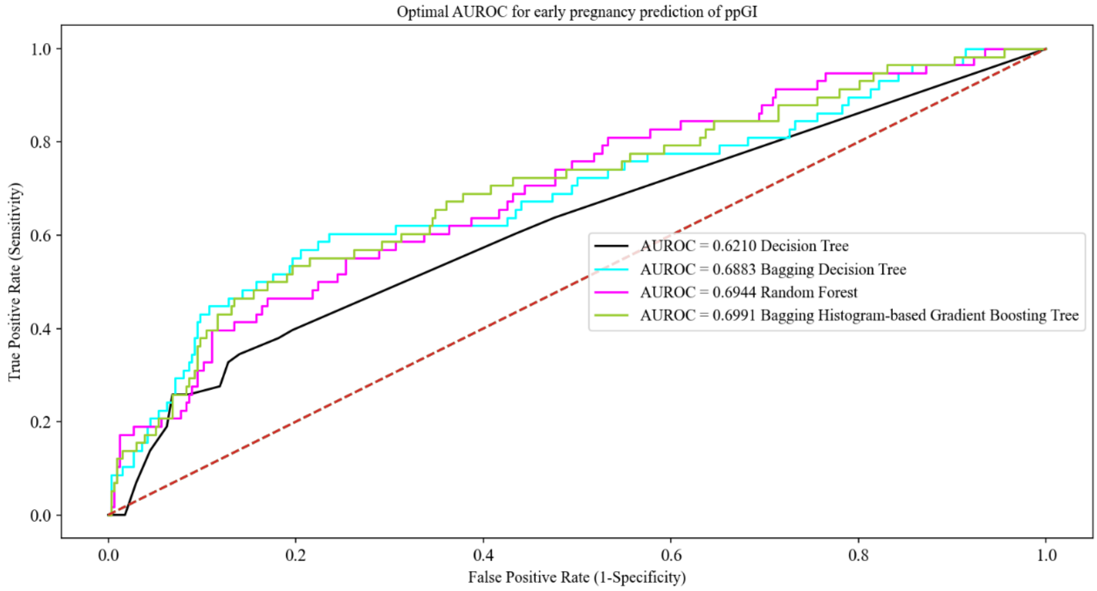

Fig S5: Variation in the mean stratified 4-fold cross validation accuracy as a function of the lasso regularization hyperparameter C for the final model. Maximum CV-accuracy of 0. 8554 is obtained for C=0.0798, related to Figure 2.

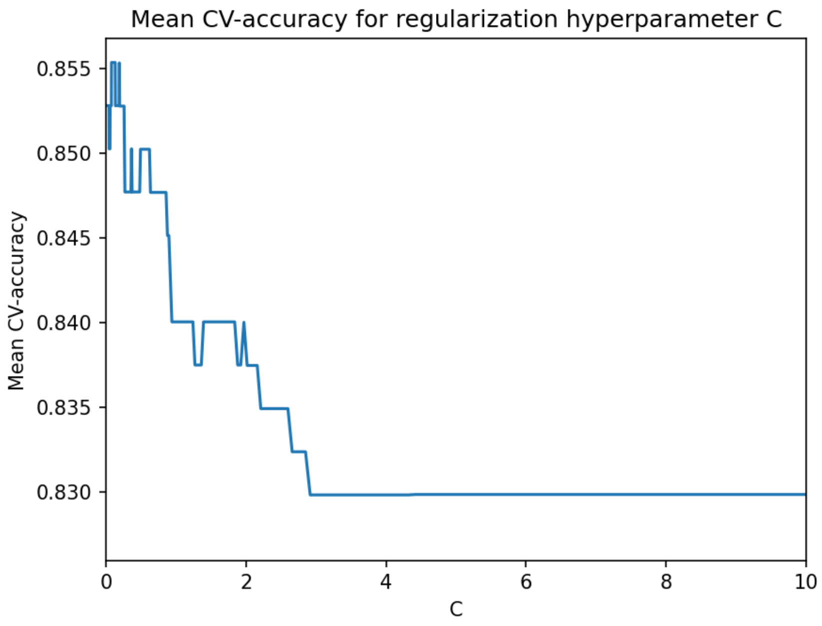

Fig S6: ROC curve for early ppIFG prediction using LR, related to Figure 2.

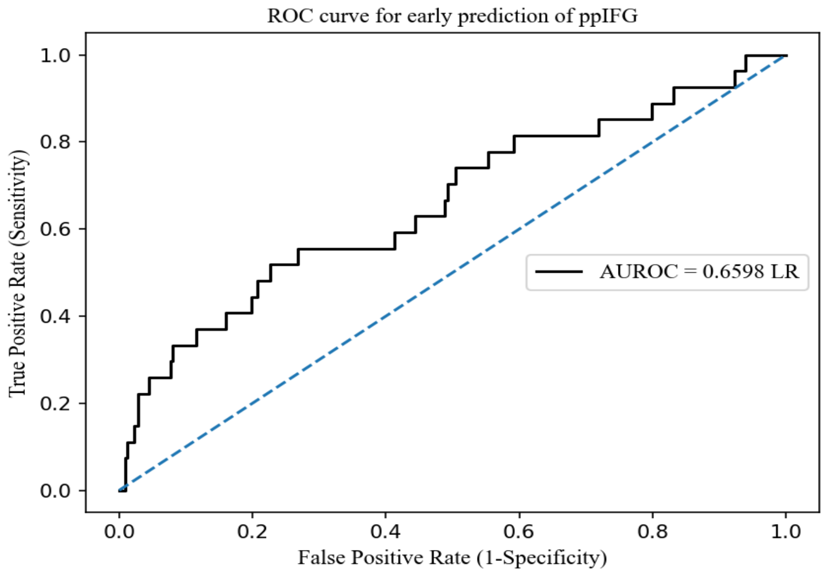

SHAP Summary plots

Fig S7: Decision tree model SHAP summary plot, related to Table 4.

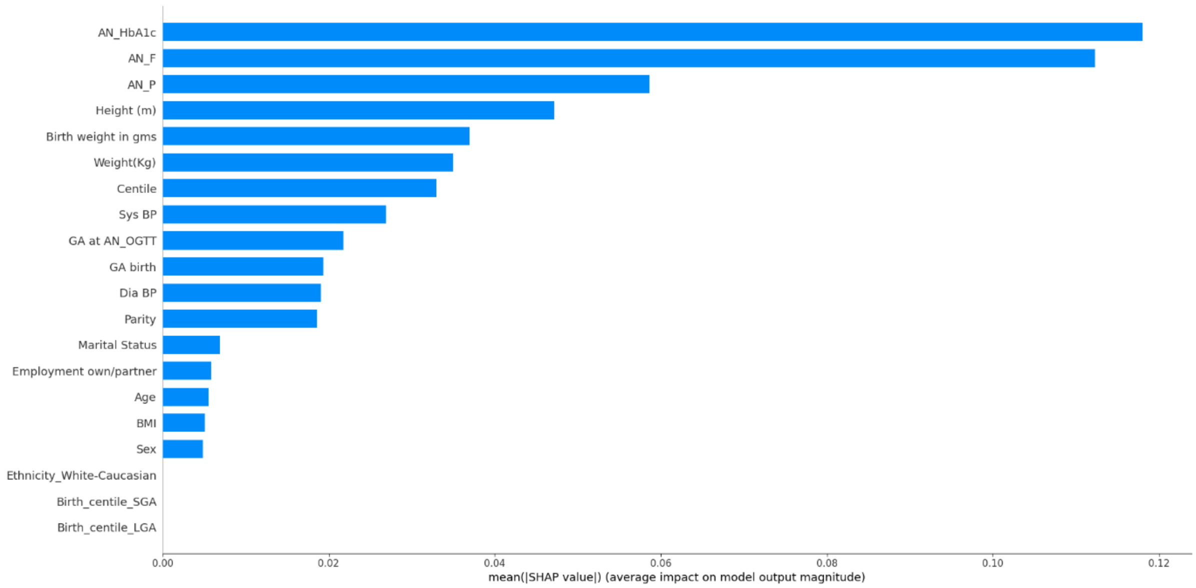

Fig S8: lightGBM model SHAP summary plot, related to Table 4.

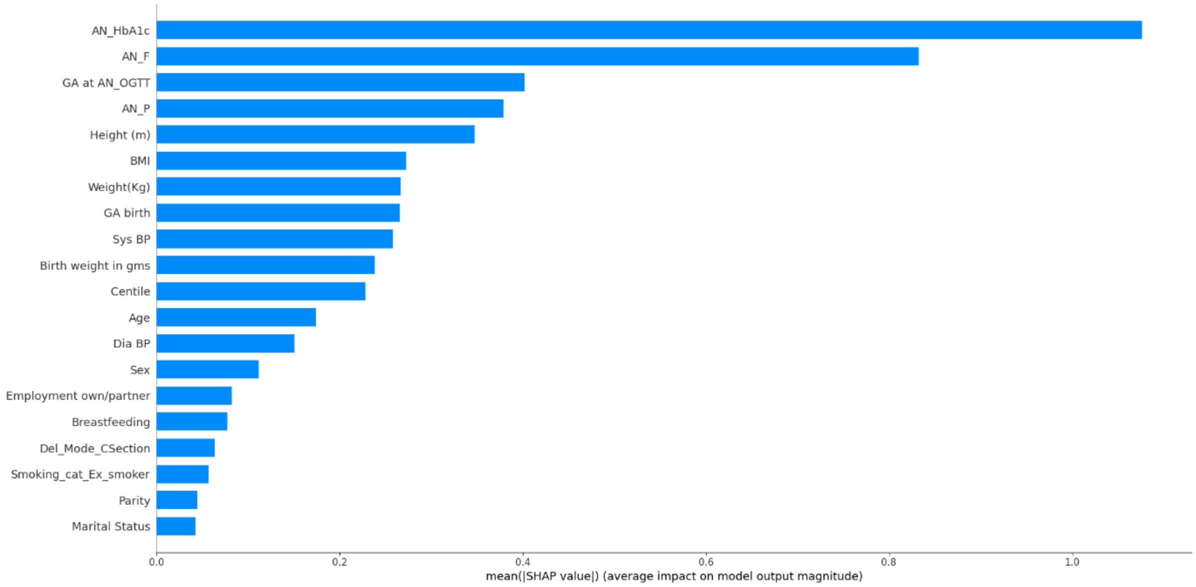

Fig S9: Calibration plot of the selected LR model, related to Figure 2.

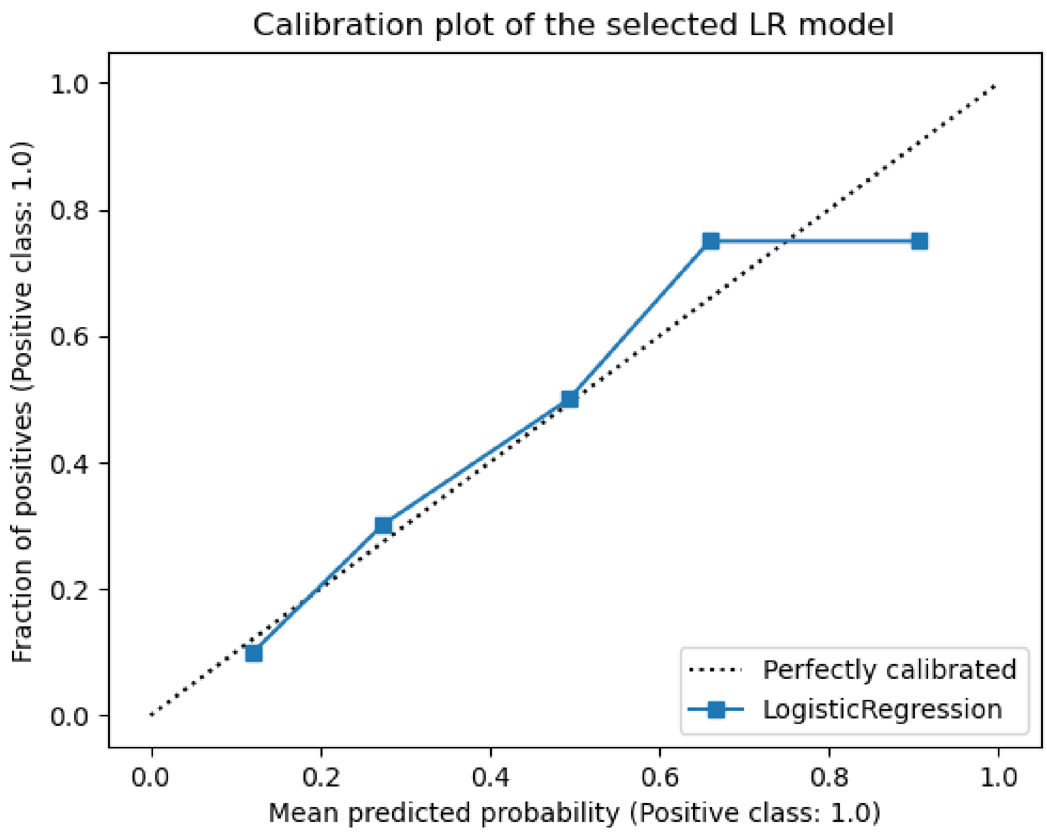

Fig S10: Standard letter format for postpartum screening to all GDM women issued from the Diabetes clinic, GEH-NHS Trust, UK, related to Figure 1.

George Eliot Hospital
NHS Trust

George Eliot Hospital
College Street
Nuneaton CV10 7DJ
Telephone: (02476) 351351
Facsimile: (02476) 865175

Dear Madam

Gestational Diabetes Post-Natal Blood Test

You may remember during your recent pregnancy, you developed gestational diabetes. We recommend that you have a blood test following delivery of your baby to ensure that your blood glucose levels have returned to normal. Based on your results during pregnancy we advise that you have the following test:

- ☐ Glucose Tolerance Test- 6-12 weeks post delivery
- ☐ HbA1C- 3 months post delivery

Please find enclosed the relevant blood form.

Please ensure you make an appointment with your GP or practice nurse to obtain your results and further advice.

In addition, the following advice for the future is recommended:

1. If you plan to conceive or become pregnant, then be aware that you can develop diabetes again during pregnancy. If this is the case please make arrangements to visit your midwife or doctor immediately as you will require further tests.

2. You also have an increased risk of developing diabetes in the future and we advise you to maintain a healthy diet and lifestyle. Please see enclosed leaflet.

3. Due to this increased risk of developing diabetes, we also recommend you have a yearly HbA1C blood test.

If you develop any symptoms of diabetes you should be tested sooner and see your doctor immediately. These symptoms can include increased tiredness, thirst, and frequency in passing urine, blurred vision and thrush type infections. If you experience any of these or are in any doubt please see your doctor or nurse.

Yours sincerely

Prof Saravanan	J Plester	J Wilson / S Selvamoni	T Ritchie
Consultant	Diabetes Midwife	Diabetes Specialist Nurses	Diabetes Associate Practitioner
